# Sense of Belonging, Meaningful Daily Life Participation, and Well-Being: Integrated Investigation

**DOI:** 10.3390/ijerph20054121

**Published:** 2023-02-25

**Authors:** Dorit Haim-Litevsky, Reut Komemi, Lena Lipskaya-Velikovsky

**Affiliations:** 1 Beer-Yaakov-Ness-Ziona Mental Health Center, Beer Yaakov 70350, Israel; 2 School of Occupational Therapy, Faculty of Medicine, The Hebrew University, Jerusalem 91904, Israel

**Keywords:** healthy population, participation diversity, daily life occupations, community belonging

## Abstract

The association between well-being, sense of belonging, connectedness to community, and meaningful participation in daily life occupations was theoretically proved and demonstrated in several health conditions or specific age groups. This study aimed to investigate an interplay between well-being, sense of belonging, and connectedness, and meaningful participation in a range of daily life occupations among healthy adults of working age in Israel. Participants (N = 121; age: M = 30.8, SD = 10.1; women: N = 94, 77.7%) completed standard instruments to evaluate the main constructs through an internet survey. A variety of communities, that the participants reported to belong to, were not different in the sense of belonging and connectedness, participation dimensions, and well-being. An association was found between sense of belonging and connectedness, the participation subjective dimension, and well-being (0.18 < r_p_ < 0.47, *p* < 0.05). Sense of belonging explained in a significant way the variance in well-being (F(3) = 14.7, *p* < 0.001; R^2^ = 0.274) and was found to be a mediator between participation and well-being (1.86 < Sobel test < 2.39, *p* < 0.05). The study provides empirical support to the interrelationship between meaningful participation, sense of belonging and connectedness, and well-being in a healthy population. Participation in a range of meaningful activities that contribute to the sense of belonging and connectedness as a universal concept could further promote well-being.

## 1. Introduction

### 1.1. Literature Review

Well-being is closely related to individual and community health, flourishing, and prosperity [1,2,3,4]. An in-depth understanding of inherent mechanisms underlying well-being will contribute to our insights on this complex phenomenon and delineate venues for promotion. There is a theoretical underpinning on an interplay between well-being, meaningful participation in a range of daily life occupations, and sense of belonging and connectedness to community [5,6,7,8,9,10]. However, the integrated research on such an association is in its infancy, and was limited to specific health conditions, age groups, or types of occupations. This work addresses this gap by investigating the interrelationship between these three constructs in a healthy population within an age range. 

#### 1.1.1. Well-Being

Well-being is a multidimensional construct that refers, in general, to a state of overall contentment and harmony [1]. Being under ongoing development, it was conceptualized through a different lens by a range of models from various disciplines [3]. Well-being can be defined from an objective perspective through quantifiable social or economic indicators (such as income levels, living conditions, leisure time, and life expectancy) [3,4]. However, the most common models define well-being through subjective aspects (including the presence of positive emotions, low negative emotions, and satisfaction with life) and psychological aspects (including environmental mastery, self-acceptance, meaning and purpose in life, positive relationships with others, autonomy, and personal growth) [11,12,13,14,15]. Traditionally, subjective well-being is placed in the context of ‘hedonic’ vision, or the experience of pleasure and happiness, including both an affective component concerned with emotions and a cognitive component of how people evaluate their own lives [14,15]. This is compared to psychological well-being, which is guided by ‘eudaimonic’ vision—striving for optimal psychological functioning, and pursuing meaning and self-realization [11,15,16]. In spite of the two streams of the operationalization of well-being, a multitude of related and overlapping terms were used in the literature [4,16,17]. Subjective well-being is often used interchangeably with mental well-being, mental health, and even psychological well-being and happiness [17]. This theoretical ambiguity is reflected in measuring procedures, while the evaluation of the well-being within both streams commonly addresses the following dimensions: mental, social, physical, and spiritual well-being, activities and functioning, and personal circumstances [1]. Recently, it was argued that there are additional aspects that are of prominent interplay with well-being that should be addressed: social equality, local culture, environment features and properness, governance structure, and political freedom [3,18].

Based on the World Health Organization (WHO) definition, well-being reflects a state of health with blurred boundaries between the concepts [2]. For example, it was found that higher well-being reduces a risk for development of mental illness, is predictive of recovery from physical and mental illness, reduces health-risky behaviors, and increases life expectancy [3,17]. Given its importance, well-being became a focus for concern, research, and action in recent decades [1,2,3,4].

#### 1.1.2. Community Connectedness and Belonging

One of the important social determinants of well-being is a sense of community connectedness and belonging [1,19,20,21,22,23]. Social connectedness and sense of belonging—the feelings of being a part of a larger group of individuals—are thought to be basic human needs [24,25]. The experience of a significant bond with a physical, relational, symbolic, or even imagined collectives is crucial for the survival of the individual, as well as for the continuity and prosperity of the community [26,27]. Even though there are several models to conceptualize the sense of connectedness and belonging, most of them acknowledge identical key aspects [5,6]. An individual’s sense of social connectedness and belonging is based on the perception of how she/he relates to others or a community, with an emphasis on the relationship’s quality and the nature of mutual trust and reciprocity; these over and above practical and technical aspects of social support [3,4,19,20,28,29]. The prevalent theoretical framework for a sense of community addresses four core elements [6]. ‘Membership’ (1) addresses feelings of belonging, emotional security, and identification. ‘Influence’ (2) refers to the ability of members make an impact on a community, and vice versa. ‘Integration and fulfillment of needs’ (3) implies that the community is capable of satisfying the physical and psychological needs of its members, which will reinforce members’ commitment to it. ‘Shared emotional connection’ (4) stems from identifying with a shared history of the community through personal investment and interaction with other members of the community [6,24]. In such a way, a sense of connectedness and belonging reflects a subjective evaluation of the extent to which one has meaningful, close, and constructive relationships with others (i.e., individuals, groups, and society) and it may be easily understood as an experience opposite to loneliness [29,30,31]. The importance of social connectedness and sense of belonging is supported by the findings on numerous health risks of the experience of social isolation and loneliness, including a suicide attempt, self-harm, higher prevalence of mental health disorders, exposure to violence, poor physical health, substance use, etc. [6,20,28,32,33]. The hurtful effects of an interruption in social connectedness and belonging on health were found to be comparable to the risk of well-established health risk factors, such as obesity or smoking [33]. It was found that personal factors, such as individual social skills and cognition, with their developmental trajectory and processes of acquisition, contribute to the experience of social connectedness and belonging [34]. Still, the role of community and environment is crucial for the development and continuity of the experience [22,30,35,36]. The profound effect of the sense of social connectedness and belonging on well-being and health with the potential to improve the outcomes [25,30] became a focus of health services, social and political institutions, and research in recent years [23]. To date, social connectedness and belonging was investigated moslty in context of specific predefined communities [6,30,37]. Moreover, given the potential impact of culture and socio-political factors on both well-being and a sense of connectedness and belonging, it is important to investigate this interplay in different communities.

#### 1.1.3. Participation

Participation in meaningful daily life activities was suggested to be one of the mechanisms fostering social connectedness and belonging and vice versa [5,6,10,26,30,38]. The World Health Organization (WHO) defines participation as “involvement in a life situation” [2]. Within specific literature, participation is defined as a naturally occurring, active engagement in purposeful and meaningful occupations of daily life [39,40]. Daily life occupations encompass a range of areas, including “all individual’s pursuits; mental, physical, social and spiritual; restful, reflective and active; obligatory and self-chosen; paid and unpaid” [41]. Participation comprises objective aspects (e.g., diversity of activities and intensity or frequency of participation) and subjective aspects of individuals’ experiences [39]. It occurs as the result of choice, motivation, and meaning within a supportive context, and contributes to development of mastery, identity, and social and environmental interactions [40,42]. Indeed, mutual relationships between individuals’ occupations and personal experiences within social environments and relationships were identified already in the early stages of the formation of the concepts of community belonging and connectedness [21] and were further supported through the research [43]. It was found that participation in social activities is a pre-requisite for the maintenance of meaningful relationships, a means to realize the relationship, and a valuable indicator of connectedness and sense of belonging [24,25,36,43,44,45,46]. The occupational perspective acknowledges this interrelationship, arguing that through occupations, individuals communicate with others and interface with the environment [7,8,9,39,40,47]. The results of human occupations are brought into the community, being a way both to express connectedness and belonging and to experience them [7,8,9,48]. Personal occupations are deeply influenced by community values; they may be driven or hindered by the meaning that the community ascribes to the occupations and feedback provided by others upon performing the occupations [7,8,9,48]. This interrelationship is so tight that it was argued within occupational science that belonging and connectedness are integral dimensions of occupations [7]. There is an empirical base for the association between a sense of belonging and connectedness and participation in civil and political community activities that was demonstrated in different countries, such as Australia, the USA, and Italy [26,43,49,50], yet the strength of the relation between the concepts is still unclear [26]; moreover, a wide range of participating occupations was negligibly addressed, till now, within the context of belonging and connectedness [26]. Most importantly, both participation in daily life activities and a sense of connectedness and belonging are thought to be basic human rights contributing to social justice and equity [27,42].

#### 1.1.4. Well-Being, Participation, Connectedness, and Belonging

Similar to the sense of community connectedness and belonging, participation in meaningful occupations appears to be an important component of well-being [1,7,9,48]. These imply, in a natural way, the importance of interplay between participation in meaningful activities and a sense of connectedness and belonging for well-being and health. Indeed, the theoretical roots of the interrelations between these three constructs—participation, belonging and connectedness, and well-being—may be found in both belonging and connectedness models [5,6], as well as in occupational science [7,8,9,10]. Moreover, this interplay receives intensive support from general theories, such as self-determination theory, being conceptualized through integration between three basic concepts: autonomy, competence, and relatedness as prerequisites for well-being and health [51]. Still, further empirical support is needed to expand our understanding of this interplay.

Research that focuses on the association between participation, belonging, connectedness, and well-being addressed clinical populations of cancer survivors [52], individuals with dementia [53], cerebral vascular accident (CVA) [54], and serious mental illness [55,56]. Following theoretical assumptions, these studies demonstrated the interplay between the constructs, revealing their complex nature. For example, the findings on cancer survivors demonstrated that participation in daily life occupations that are not social by their practice but contribute to the lives of others and family life-related occupations were associated with a sense of belonging and contribute to subjective experiences of well-being, in addition to social occupations with wider social networks [52]. In healthy populations until now, the research mostly involved an elderly population or adolescents [45,57] and focused on the investigation of participation in social activities, both formal and informal [45,58], and community-oriented activities, such as volunteering [59,60], as contributing to health and well-being. However, participation in a range of occupations and activities may contain aspects of a sense of connectedness and belonging and contributes to it. For example, preparing meals happens frequently in a non-social environment, but its contribution to connectedness and belonging may be substantial if they are prepared for family or friends. Thus, the research should address the whole landscape of individual participation. The interplay between a sense of connectedness and belonging, range of participation in meaningful daily life activities, and well-being were little investigated in an adult, healthy population in general, and in different cultures in particular [43]. This information can expand our knowledge of inherent mechanisms underlying each one of the constructs and may suggest a pathway to enhance the health and well-being of individuals and community prosperity through connectedness, belonging, and participation. Moreover, knowledge of the interplay between these constructs in health has the potential to advance an understanding of the impact of medical conditions on this interplay, and again, may delineate venues for change in vulnerable populations.

### 1.2. Study Aims and Hypotheses

This study aimed to investigate quantitatively an association between well-being, sense of belonging and connectedness, and objective and subjective dimensions of meaningful participation in a range of daily life occupations, apart from social and political ones, among healthy adults within a span of working age in Israel. We hypothesize that indices of participation in a range of daily life activities and a sense of belonging and connectedness will be associated. In addition, we assume that the mediation effect will be found between the participation, sense of belonging and connectedness, and their association with well-being.

## 2. Materials and Methods

This is a cross-sectional study with convenience sampling based on volunteers, recruited through social internet platforms from February to May 2018.

### 2.1. Participants

The inclusion criteria for this study were adults of working age (for women: 18–62; for men: 18–67) and healthy according to their self-report. The operational definition of “health” for this study was as follows: personal experience of health that does not limit any aspect of their life; no known medical diagnosis; and no regular medications besides food supplements, such as vitamins. People who did not report on a sense of belonging to any community and people, who experienced immigration in the previous 5 years, or had a low literacy level, were excluded from the study. The sample size was calculated using G*Power, V3.1 software based on the previous research on the association of sense of belonging as measured with SCI and well-being in the healthy population [60]. Based on the reported association of r = 0.32, α = 0.05, and power of 0.8 (two-tailed assumption), the sample size was found to be N = 74. Due to the methodology of the data collection using a non-commercial internet survey, with limited real-time control for participants’ characteristics, the final recruitment sample size was doubled.

One hundred fifty-nine volunteers participated in an internet survey. After providing informed consent to participate in the study, 26 (16.3%) participants reported no sense of belonging to any community, and 12 (7.5%) reported themselves as not healthy; thus, they did not continue with the research procedures. One hundred twenty-one volunteers completed the study procedures. They were aged 19–63 (mean = 30.84, SD = 10.1), mostly women (women: N = 94, 77.7%; men: N = 27, 22.3%), native-born (N = 101, 83.5%), working (N = 59, 48.8%) or studying (N = 58, 47.9%), living in an urban environment (N = 96, 79.3%), and had lower than average income (lower than average: N = 78, 64.5%; average income: N = 29, 16.5%; higher than average: N = 23, 19%). People who were not native-born (N = 20, 16%) were living for at least 7 years in the same country (range: 7–28). The following family statuses were reported by the participants: single (N = 66, 54.5%), married (N = 48, 39.7%), divorced (N = 4, 3.3%), and not interested in reporting on family status (N = 3, 2.5%). Most participants had high education (high education: N = 93, 76.9%; secondary education: N = 25, 20.7%; and incomplete high-school education: N = 3, 2.5%). The sample of the study is representative of the Israeli population as for the geographic location, type of residence, and income, but not for the family status and level of education [61].

### 2.2. Measurements

The Satisfaction with Life Scale (SWLS) [62] was used as a proxy measure of well-being for an otherwise healthy population. SWLS is a self-reported tool with five statements, rated by the person on a 7-point Likert scale for the agreement (1 = “strongly opposes” to 7 = “strongly agrees”). The overall score of the questionnaire is a sum of the ratings, with the possible range of scores being 5–35. A score of 20 points represented a neutral approach. Scores between 5 and 9 indicate that the subject was very dissatisfied with his life, while scores between 31 and 35 indicate that the subject was very satisfied. Internal consistency of the questionnaire is sufficient (0.79 < Cronbach’s α < 0.89). The test–retest reliability (0.80 < α < 0.84) and construct validity were demonstrated for the tool [62].

The Sense of Belonging Instrument (SOBI) [29] is a self-reported questionnaire that assesses the individual’s sense of belonging. The questionnaire items are based on the Hagerty model of belonging and addresses the psychological measure of a sense of belonging (valuable involvement with a focus on personal experience) and the prerequisites for a sense of belonging (energy, desire, and potential for valuable involvement). We used only the psychological index, which contains 18 items. The items are scored on a 4-point Likert scale (1 = strongly disagree, and up to 4 = strongly agree). The total score is calculated by average, with a higher score indicating a greater sense of belonging. The questionnaire was found to have a medium-high internal consistency (0.72 < Cronbach’s α < 0.76) and high test–retest reliability (0.66 < Cronbach’s α < 0.84). Content validity was determined by seven experts who determined if each item was relevant to the definition of the term [29].

The Sense of Community Index (SCI-2) [31] is a self-report questionnaire that is widely used for measuring a sense of community belonging with a focus on general attitudes and approaches. The questionnaire consists of 25 items and is organized into 4 sub-scales: reinforcement of needs, membership, influence, and shared emotional connection (6 items for each sub-scale), and 1 general question. The sub-scales’ items are rated with a 4-point Likert scale (1 = “not at all” to 4 = “always”). The total score of each sub-scale is a mean of the relevant items’ ratings and the total questionnaire score is a sum of 25 items. The higher the score, the higher the person’s sense of belonging to the community. There is an internal consistency of the SCI-2 total score (Cronbach’s α = 0.94), as well as for the sub-scales (0.84 > α > 0.79). The SCI-2 was found to be valid and highly predictive for social behavior [31].

The Meaningful Activity Participation Assessment (MAPA) [63] is a subjective measure of the degree of meaning in the participated activities. The tool includes a list of 28 occupations/activities. The person is asked to report the frequency of participation in each activity (0 = not at all, 6 = every day) and the level of personal importance—meaning—for each item (0 = not important at all, 4 = very important). Several indices may be calculated for the tool: the number of participated activities—participation diversity, the frequency of participation—participation intensity, and meaningfulness of the participation (calculated by the sum of the importance score multiplied by the participation rating for each item). The maximum score of meaningfulness is 672. The higher score indicates a greater perception of the meaning of the activities in which the person participates. The questionnaire is reliable and valid: internal consistency (Cronbach’s α = 0.85), test–retest reliability (r = 0.84, *p* < 0.01), and construct validity were reported [63].

#### Reliability of the Measurements

Internal consistency of the scales was calculated for the study sample and was found to be sufficient for SWLS (Cronbach’s α = 0.859), SOBI (Cronbach’s α = 0.849), SCI-2 (total score: Cronbach’s α = 0.94; reinforcement of needs: Cronbach’s α = 0.79; membership: Cronbach’s α = 0.72; influence: Cronbach’s α = 0.85; shared emotional connection: Cronbach’s α = 0.89), and MAPA (reports on meaning: Cronbach’s α = 0.86; participation frequency: Cronbach’s α = 0.73) scores.

### 2.3. Procedures

The study was carried out according to the Declaration of Helsinki and was approved by the Institutional Review Board. The survey was distributed through non-formal internet social networks of local information on Facebook in three different geographic areas (one central and two peripheral). The advertisement of the study contained general information on its purpose and procedures, including volunteer-based participation and a link to the study’s webpage. First, all the participants who chose to participate in the study provided electronically informed consent after being presented with additional information on the study’s aims and procedures. Only for those who provided informed consent was the electronic survey open for completion through either a personal computer or mobile device. The survey included a demographic questionnaire, followed by the self-administrated tools in random order: the SWLS, the SCI-2, the SOBI, and the MAPA. Participants who did not meet the inclusion criteria based on the demographic data were discontinued automatically with the study procedures.

### 2.4. Data Analysis

Descriptive statistics were used to characterize the study participants and main study variables. The type of distribution of the study variables was investigated with the Kolmogorov–Smirnov test and was found to be different from normal for all the measurements, except for MAPA intensity and total scores. The reliability of the tools with the study cohort was calculated using Cronbach’s α. Association between the measurements was estimated with the Pearson correlational coefficient based on sample size or with χ^2^ test. Between-group differences were analyzed based on the data distribution as follows: Between-gender and age-related differences were analyzed using the t-test and Mann–Whitney test. Differences between various types of communities were investigated using one-way ANOVA and the Kruskal–Wallis test. Since the groups were not equal as to the number of participants, we calculated effect size (ES, η^2^) to support the findings in the between-groups differences. Linear regression with the enter method was used as multivariate analysis to investigate the unique contribution of years of education, participation dimensions, and connectedness and belonging indices to explain the variance in well-being. The Sobel test was used to assess the mediation effect. The data were analyzed with SPSS, 27, and the significance level was set at 0.05 for all statistical tests.

## 3. Results

### 3.1. Descriptive Statistics

The study participants reported on a sense of belonging to various communities as detailed in Table 1. Descriptive statistics of the main study variables are presented in Table 2. The overall experience of a sense of belonging, based on SOBI results, was relatively higher for most of the participants, while their report on the sense of community connectedness (SCI-2) greatly varied (Table 2). Half of the people who took part in the study participated in 78.6% of the addressed activities (22 from a total of 28), with a variable frequency and a wide range of experienced meaning in the participated activities (Table 2). In general, the experience of well-being was positive for around half of the participants (Table 2).

We performed an analysis of the main study indices by groups based on demographic data. No statistical differences were found between men and women in all indices of a community of belonging or in communities for belonging (χ^2^(5) = 5.22, *p* > 0.05). Still, it was a trend for women to experience more frequently a sense of belonging to a religious community, whereas men felt belonging to leisure groups (Table 1). No differences were found between genders in the satisfaction with life, participation diversity, and meaningfulness of participation; however, a difference was found in the participation intensity in favor of women (Table 3).

No association was found between age and main variables of belonging, participation, and satisfaction with life (−0.098 < r_p_ < 0.04, and *p* > 0.05). The data were split by the age groups: young adults (age 19–27: N = 68; 56.2%) and adults (age 28–63: N = 53, 43.8%). The analysis yielded no statistically significant differences between the age groups in all the study variables (Table 3). Still, the correlation was found between years of education and the SOBI (r_p_ = 0.2, *p* < 0.05) and SWLS (r_p_ = 0.18, *p* > 0.05), but not with other scales (−0.07 < r_p_ < 0.03, *p* > 0.05). No differences were found in the main variables of interest between groups by type of settlement (urban versus non-urban) (SCI-2: U = 987.5, *p* > 0.05, η^2^ = 0.02; SOBI: U = 1193.5, *p* > 0.05, η^2^ = 0.001; MAPA diversity: U = 1182, *p* > 0.05, η^2^ = 0.001, intensity: t(119) = 0.95, *p* > 0.05, η^2^ = 0.01, total: t(119) = −0.7, *p* > 0.05, η^2^ = 0.004 and SWL: U = 1142, *p* > 0.05; η^2^ = 0.001) and family status (SCI-2: H = 1.5, *p* > 0.05, η^2^ = 0.009; SOBI: H = 4.3, *p* > 0.05, η^2^ = 0.03; MAPA diversity: H = 2.3, *p* > 0.05, η^2^ = 0.02, intensity: F(2115) = 1.1, *p* > 0.05, η^2^ = 0.02; total: F(2115) = 0.8, *p* > 0.05, η^2^ = 0.014 and SWL: H = 4.5, and *p* > 0.05; η^2^ = 0.03).

### 3.2. Well-Being, Sense of Belonging, and Meaningful Participation among Study Participants

The correlation analysis indicates a significant and moderate association between well-being (SWLS) and the parameters of sense of belonging and connectedness (SOBI and SCI-2) (Table 4). In addition, a significant, still weak correlation was found between well-being and experience of meaning in participated occupations. No association was found between satisfaction with life and participation diversity and intensity (Table 4). The higher sense of belonging and the higher level of meaningful participation were congruent with the higher level of well-being. A significant moderate correlation was found between the experience of meaning in participated occupations and sense of belonging measurements with congruence (Table 4). However, a significant, but weak, correlation or no correlation was found between the participation diversity, intensity, and sense of belonging (Table 4).

Next, the results were organized by the type of community of belonging. No statistical differences were found between the groups in well-being, participation diversity, intensity, and meaningful participation, and the sense of belonging indices, except for the shared emotional connection (Table 5). Based on effect size parameters, no trend in difference was detected between the groups in the main variables of interest in the study.

Regression analysis indicated a significant contribution of years of education to the explanation of the experience of well-being (SWLS) in the first model (3.2% of explained variance). This impact was overcome by the contribution of the meaningful participation index (MAPA) in the second model with significant improvement in the explained variance (Table 6). The second model explained 10% of the variance in the SWLS score. The third regression model included the SCI-2 index in addition to the years of education and MAPA. It was found to be significant, explaining 22% of the variance and all the independent variables (years of education, the MAPA, and the SCI-2) contributed significantly to the explanation (Table 6). However, the higher explanation of 29% was obtained through the fourth model, which included years of education, the MAPA, SCI-2, and SOBI scores (Table 6). However, the contribution of the MAPA score and years of education to well-being was overcome in the fourth stage of the analysis by the indices of sense of belonging and connectedness (SCI-2 and SOBI).

**Figure 1:**
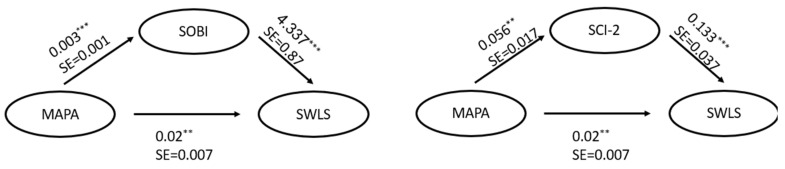
Mediation effects: sense of connectedness and belonging. Note: ** *p* < 0.01; *** *p* < 0.001; SCI-2—Sense of Community Index, 2; SOBI—Sense of Belonging Instrument; MAPA—Meaningful Activity Participation Assessment; and SWLS—Satisfaction with Life Scale.

Based on our hypothesis and the results of the regression analysis, we investigated the mediation effect of a sense of belonging and connectedness on the association between meaningful participation and well-being. The SOBI and SCI-2 scores both were found to be significant mediators between meaningful participation and well-being (Sobel test = 3.03, *p* < 0.01; Sobel test = 2.42, *p* < 0.01, correspondently) (Figure 1).

## 4. Discussion

This study aimed to investigate the interplay between three constructs: a sense of belonging and connectedness, meaningful participation in a range of daily life occupations, and well-being among an otherwise healthy population to understand the phenomenon. The study provides empirical support to the premise of the interrelationship between these three constructs [5,6,7,8,9,10,30], demonstrating an association between each pair of the constructs. Moreover, the study contributes to a more nuanced understanding of the complex interplay between the constructs, elucidating a mediating effect of a sense of belonging and connectedness on the association between meaningful daily life participation and well-being.

People experience a sense of belonging and connectedness to a variety of communities [30]. Indeed, there was a wide range of reported communities to belong to in this study, e.g., family, work, leisure groups, etc. Each type of community had its specific characteristics, suggesting different social roles, patterns of relationship, and communications, and providing different pathways and possibilities for connectedness and belonging [64,65]. Following that, the assumption was that the discrepancy in the experience of connectedness and belonging would be found between different communities. This assumption was supported by the findings from the previous studies, which investigated different types of communities to belong to, such as a sport or choir [30,66,67,68,69]. Interestingly, while comparing the range of communities to belong to, we found no difference in the indices of belonging and connectedness between them. The findings support the notion that the experience of connectedness and belonging is a universal phenomenon and may be achieved for different communities through various pathways. This result is of importance in light of the strong association between the extent of the sense of connectedness and belonging and the level of well-being [23,25,30], suggesting that once the person established connectedness and belonging to any community, this provides a beneficial effect on well-being.

The study participants were recruited regardless of a specific social context and were offered an opportunity to define the community they address through the study. To our vision, the results demonstrate the importance of personal choice and freedom in the process of defining the community to which one feels a connection and belonging beyond practical aspects of acting in those or another community. Moreover, the experience of choice may be an enabling factor that mitigates the gap between community characteristics and demands, and individual skills and needs. The reciprocity between people and community supports the development of belonging and connectedness, impacts its sustainability, and contributes to the individual experience of well-being [1,19,20,21,22,23]. Moreover, the experience of choice, as well as experience of belonging and connectedness, addresses mechanisms of meaning and purpose in life, positive relationships with others, autonomy, and personal growth, while all of them have the potential to further contribute to psychological aspects of well-being [11,12,13,14,15].

Understanding factors that are supportive of the sense of connectedness and belonging is imperative. Following the previous studies [23], we found no difference in the sense of connectedness and belonging between genders and different age groups. Surprisingly, and contradictory to the literature [5,44,70], we found no difference in connectedness and belonging between various types of settlements, while addressing a range of communities to belong to. Little differences in the sense of belonging and connectedness may mirror the universality of belonging and connectedness, as was previously suggested [71]. Still, following previous research [72], we found an association between the level of education and sense of community belonging and connectedness, implying the importance of demographic factors and requiring further investigation of their impact.

The notion of the universality of belonging and connectedness was further supported by additional findings on the similarities in a range of participated-in activities, the frequency of participation, and the experience of meaning in the participated-in activities among different communities. Naturally, differences in the participation patterns can be posited between different communities of belonging, since, for example, belonging to a religious or leisure community supposedly means engagement in different activities, mostly additional to the basic daily life ones, and has the potential to bring about a different level of enjoyment or meaning [39]. However, our findings refute this premise.

We demonstrated a link between the subjective dimension of meaningful participation in a range of daily life activities and a sense of community belonging and connectedness in an otherwise healthy population. These findings confirm the results of studies with various clinical populations [52,53,54,55], supporting the theoretical foundations of occupational science [7,8,9,10] and the assumptions of belonging and connectedness models [5]. Indeed, the very performance of occupations, which involve another person in some way, appears to be by its nature an indicator of a significant connection [7]. The findings of this study are particularly intriguing since we measured participation in common, non-social, and social daily life occupations in all the communities of belonging to enable comparison between them. The results may suggest that connectedness and belonging, as a universal phenomenon, have a beneficial effect on the whole experience of participation in a scope of activities, even those that do not relate directly to the roles in the community of belonging. In addition, the congruency between the extent of meaningful participation and the experience of connectedness and belonging implies the importance of the meaning, which the community ascribes to occupations. Still, the association between the constructs was modest. Such strength of correlation reflects aspects of the uniqueness of each construct, albeit the convergence, further supporting the notion of the complex nature of both constructs. Meaningful participation in daily life activities and a sense of belonging and connectedness relies naturally on multiple, sometimes detached, factors [39,72]. On one hand, indices of both meaningful participation and connectedness and belonging emphasize individual experience and feelings over and above practical and technical aspects, implying in such a way congruency between them [3,4,19,20,28,29]. On the other hand, participation in specific occupations may be driven by additional individual needs other than community connectedness and belonging, such as a need for health safety when a person communicates with his doctor on healthcare issues [73]; or, a sense of belonging and connectedness may be not achieved through participation in various activities, even social ones, as in the case of having a holiday dinner with family during a long-lasting family conflict. Next, the findings suggest that in the healthy population, the level of sense of connectedness and belonging intervene in a limited way with the objective aspects of participation in common daily life occupations, as little association was found between the indices. In this way, the results figure out an interplay between subjective and objective dimensions of participation, with connectedness and belonging and help in the understanding of controversy in the previous studies addressing such an association through various objective and subjective indices [52,53,54,55,66,67,68,69]. Next, subjective, but not objective, indices of the participation were found to be related to well-being, strengthening the importance of this dimension of participation. However, the results may stem from the conceptual issues. The objective participation dimension addresses objective well-being aspects that were not approached in this study, while subjective participation dimension reflects psychological well-being of meaningfulness, purposefulness, autonomy, and personal growth. Thus, the consistency of the association between subjective indices in the study within all three constructs may be less indicative of the whole phenomenon of the interrelation between well-being, participation, connectedness, and belonging, representing the congruency of subjective experience, as was previously demonstrated [74].

The paired association between the main study constructs provided a basis for further investigation of the complex interplay between a sense of community belonging and connectedness and meaningful participation in well-being. Even though the extent of meaningful participation explained in a significant way the variance in well-being, its impact was overcome by the indices of belonging in the integrative model. These findings provide additional support for a coincidence between meaningful participation and belonging and connectedness with regard to well-being. Indeed, we found a mediating effect of a sense of connectedness and belonging for the contribution of meaningful participation to well-being. The effect was higher for the measurement of belonging with a focus on personal experience than for the measurement of general attitudes. These findings suggest that one of the pathways of participation in meaningful activities that contribute to well-being is through sense of belonging and connectedness. In other words, the aspects of belonging and connectedness in meaningful participation and occupations may be incremental for well-being.

### Limitations

The study has several limitations. First, the sample size of the study is relatively small. Given the procedures of the data collection through an open internet survey, the control for the participants’ characteristics was limited. The representation of men versus women, family status of the participants, and levels of education was not in proportion to the general population. In addition, the representation of the different types of communities people belong to was not equal in the study, having the potential to affect the strength of the statistical analysis and interfere with the conclusions. While objective and subjective indices were collected for participation, the sense of belonging, connectedness, and well-being were evaluated only for the subjective indices. The gap between the objective and subjective parameters was previously documented [54], and may underlie the findings of this study with a trend toward a stronger connection between subjective indices. In addition, we used a generic measure of participation, addressing the most required daily life activities that do not cover the full range of a participation landscape, and thus, may be less sensitive to community-specific activities, as well as age- and gender-related differences. Finally, since communities are ever changing entities and may be substantially influenced by various factors, especially in periods of worldwide occurrence, such as COVID-19, the trends found in this study may undergo changes over time. Thus, the study should be replicated over time and in additional contexts, while addressing the limitations of this study, to provide in-depth insights on the phenomena and its development.

## 5. Conclusions

The novelty of this study is in the investigation of the interplay between indices of participation in a range of daily life activities and a sense of belonging and connectedness to a community of personal choice with regard to well-being. The study provides support for the notion that the phenomenon of belonging and connectedness is universal and appears to be a basic human need in the context of well-being and participation, regardless of the type of community one belongs to. We demonstrated quantitatively an interrelationship between subjective indices of well-being, meaningful participation, sense of belonging, and connectedness, revealing both reciprocity between each pair of constructs and a mediation effect of sense of belonging and connectedness in the association between meaningful participation and well-being among a healthy population. These findings spotlight the importance of addressing belonging and connectedness for well-being through participation and occupations. The study expands our knowledge of the inherent mechanisms underlying well-being and provides a foundation for the understanding of venues for change and the impact of health alterations on these mechanisms.

## Figures and Tables

**Table 1:** Communities of belonging (N = 121).

	Family	Work	Education	Sport and Leisure	Religion	Several Communities	Total
Total sample	58 (48%)	23 (19%)	15 (12%)	6 (5%)	8 (7%)	11 (9%)	121 (100%)
Men	14 (52%)	5 (19%)	3 (11%)	3 (11%)	-	2 (7%)	27 (100%)
Women	44 (47%)	18 (19%)	12 (13%)	3 (3%)	8 (8%)	9 (10%)	94 (100%)

**Table 2:** Descriptive statistics (N = 121).

		Median	IQR	Study Minimum–Maximum
MAPA	Diversity	22	20–24	10–28
SOBI		3.2	3–3.6	1–4
SCI-2	Total score	77	38–85	39–100
	Reinforcement of needs	3	2.67–3.3	2–4
	Membership	2.8	2.5–3.2	1.2–4
	Influence	3	2.5–3.3	1–4
	Shared emotional connection	3	2.7–3.7	1–4
SWLS		26	22–30	11–35
		Mean	SD	Study minimum–maximum
MAPA	Total score	192.1	63.2	30–357
	Intencity	3.48	0.56	2.1–6.7

Note: SCI-2—Sense of Community Index, 2; SWLS—Satisfaction with Life Scale; SOBI—Sense of Belonging Instrument; and MAPA—Meaningful Activity Participation Assessment.

**Table 3:** Sense of belonging and connectedness, participation, and satisfaction with life by gender (N = 121).

	Gender				Age Groups			
	Women (N = 94) Med (IQR)	Men (N = 27) Med (IQR)	Differences		Young Adults (N = 68) Med (IQR)	Adults (N = 53) Med (IQR)	Differences	
			U	η^2^			U	η^2^
MAPA								
Diversity	22 (20−24)	22 (19−24)	1255.5	0.000	23 (21−25)	22 (19−23)	1507	0.011
SOBI	3.2 (2.8−3.6)	3.2 (3−3.8)	1110	0.01	3.2 (3−3.6)	3.2 (2.9−3.6)	1789	0.000
SCI-2								
Total score	77 (69.75−85)	77 (65−90)	1240	0.000	77.5 (71−88)	76 (64.5−80.5)	1506	0.036
Reinforcement of needs	3 (2.7−3.3)	3 (2.7−3.5)	1261.5	0.000	3 (2.7−3.5)	3 (2.6−3.25)	1565.5	0.011
Membership	2.8 (2.5−3.2)	3 (2.5−3.3)	1206.5	0.001	2.9 (2.5−3.3)	2.8 (2.4−3.2)	1554.5	0.022
Influence	3 (2.7−3.33)	3 (2.3−3.5)	1209.5	0.001	3 (2.7−3.5)	2.8 (2.5−3.2)	1462.5	0.040
Shared emotional connection	3.2 (2.7−3.7)	3 (2.5−3.8)	1222	0.000	3.2 (2.7−3.8)	3 (2.6−3.4)	1432	0.042
SWLS	26 (22−29)	26 (23−30)	1211	0.001	27 (22−30)	26 (21.5−29)	1678.5	0.005
	M (SD)	M (SD)	t(119)	η^2^	M (SD)	M (SD)	t(119)	η^2^
MAPA Total score	192.4 (63.8)	191.3 (61.9)	0.077	0.001	200.1 (61.7)	180.1 (63.8)	1.76	0.017
Intensity	3.56 (0.57)	3.3 (0.48)	2.2 *	0.039	3.47 (0.36)	3.5 (0.66)	−0.65	0.004

Note: * *p* < 0.05; SCI-2—Sense of Community Index, 2; SWLS—Satisfaction with Life Scale; SOBI—Sense of Belonging Instrument; and MAPA—Meaningful Activity Participation Assessment.

**Table 4:** Association between study variables (N = 121).

	MAPA			SCI-2	SOBI
	Total	Diversity	Intensity		
MAPA					
Total					
Diversity	0.58 **				
Intencity	0.02	−0.41 **			
SCI-2	0.27 **	0.18 *	−0.05		
SOBI-Total	0.27 **	0.07	−0.03	0.44 **	
SWLS	0.26 **	0.05	−0.05	0.39 **	0.47 **

Note: * *p* < 0.05; ** *p* < 0.001; SCI-2—Sense of Community Index, 2; SWLS—Satisfaction with Life Scale; SOBI—Sense of Belonging Instrument; and MAPA—Meaningful Activity Participation Assessment.

**Table 5:** Study variables by the types of community of belonging (N = 121).

	Religion	Sport and Leisure	Work	Family	Education	Several Communities	Differences	
	Med (IQR)	Med (IQR)	Med (IQR)	Med (IQR)	Med (IQR)	Med (IQR)	H	η^2^
MAPA								
Diversity	23 (21–24.8)	20 (17–23)	21 (18–24)	23 (20–25)	22 (21–24)	21 (19–23)	6.2	0.05
SOBI	3.2 (2.6–3.4)	3.5 (2.7–4)	3.2 (3–3.4)	3 (2.8–3.8)	3 (3.2–3.8)	3 (3–3.4)	1.96	0.02
SCI-2								
Total score	78.5 (65.5–88.7)	81.5 (72–92)	77 (63–83)	78 (69–88)	75 (68–79)	75 (71–80)	3.47	0.02
Reinforcement of needs	3 (3–3.3)	3 (2.5–3.6)	2.8 (2.5–3.2)	3 (2.7–3.5)	3 (2.8–3.2)	3 (2.33–3.1)	2.49	0.02
Membership	2.9 (2.2–3.3)	3 (2.9–3.3)	2.8 (2.5–3.3)	3 (2.5–3.2)	2.7 (2.5–3)	2.8 (2.5–3.3)	1.96	0.01
Influence	3 (2.5–3.7)	3.2 (2.7–4)	3 (2.5–3.5)	2.9 (2.5–3.3)	2.8 (2.7–3.3)	3 (2.8–3.2)	2.8	0.02
Shared emotional Connection	3.2 (2.6–3.5)	3.3 (2.9–3.9)	2.8 (2.5–3.2)	3.2 (2.8–3.8)	2.7 (2.5–3.2)	3 (2.7–3.5)	15.2 *	0.08
SWLS	27.5 (24–30.5)	30 (24.5–34.2)	25 (22–29)	26.5 (22–29)	26 (22–30)	26 (19–29)	6.29	0.04
	M (SD)	M (SD)	M (SD)	M (SD)	M (SD)	M (SD)	F(5)	η^2^
MAPA Total score	228 (49.8)	181.5 (74.2)	178.4 (56.7)	189 (68.4)	214.7 (53.9)	185.7 (56.3)	1.2	0.05
Intensity	3.5 (0.34)	3.5 (0.48)	3.5 (0.47)	3.5 (0.66)	3.7 (0.4)	3.4 (0.46)	0.56	0.024

Note: * *p* < 0.05; SCI-2—Sense of Community Index, 2; SWLS—Satisfaction with Life Scale; SOBI—Sense of Belonging Instrument; and MAPA—Meaningful Activity Participation Assessment.

**Table 6:** Regression coefficients by the models: explanation of well-being (N = 121).

	B	SE B	β	F	R^2^	R^2^ Change	F Change
Model 1				3.99 *	0.032	-	-
Education	1.2	0.58	0.18 *				
Model 2				6.5 **	0.1	0.067	8.79 **
Education	1.1	0.56	0.17				
MAPA–total score	0.02	0.007	0.26 **				
Model 3				11 ***	0.22	0.12	18.2 ***
Education	1.3	0.53	0.2 *				
MAPA–total score	0.013	0.007	0.16 *				
SCI-2	0.15	0.035	0.36 ***				
Model 4				11.84 ***	0.29	0.07	11.32 **
Education	0.84	0.52	0.13				
MAPA–total score	0.009	0.007	0.11				
SCI-2	0.097	0.04	0.24 *				
SOBI	3.29	0.98	0.31 **				

Note: * *p* < 0.05; ** *p* < 0.01; *** *p* < 0.001; SCI-2—Sense of Community Index, 2; SOBI—Sense of Belonging Instrument; and MAPA—Meaningful Activity Participation Assessment.

## Data Availability

The data will be available upon the request from the corresponding author.
